# Brain Decoding of Multiple Subjects for Estimating Visual Information Based on a Probabilistic Generative Model

**DOI:** 10.3390/s22166148

**Published:** 2022-08-17

**Authors:** Takaaki Higashi, Keisuke Maeda, Takahiro Ogawa, Miki Haseyama

**Affiliations:** 1 Graduate School of Information Science and Technology, Hokkaido University, N-14, W-9, Kita-ku, Sapporo 060-0814, Hokkaido, Japan; 2 Faculty of Information Science and Technology, Hokkaido University, N-14, W-9, Kita-ku, Sapporo 060-0814, Hokkaido, Japan

**Keywords:** brain decoding, functional magnetic resonance imaging (fMRI), multiple subjects, visual features, generative model

## Abstract

Brain decoding is a process of decoding human cognitive contents from brain activities. However, improving the accuracy of brain decoding remains difficult due to the unique characteristics of the brain, such as the small sample size and high dimensionality of brain activities. Therefore, this paper proposes a method that effectively uses multi-subject brain activities to improve brain decoding accuracy. Specifically, we distinguish between the shared information common to multi-subject brain activities and the individual information based on each subject’s brain activities, and both types of information are used to decode human visual cognition. Both types of information are extracted as features belonging to a latent space using a probabilistic generative model. In the experiment, an publicly available dataset and five subjects were used, and the estimation accuracy was validated on the basis of a confidence score ranging from 0 to 1, and a large value indicates superiority. The proposed method achieved a confidence score of 0.867 for the best subject and an average of 0.813 for the five subjects, which was the best compared to other methods. The experimental results show that the proposed method can accurately decode visual cognition compared with other existing methods in which the shared information is not distinguished from the individual information.

## 1. Introduction

Brain decoding estimates human cognition from brain activities and has been actively studied. There has been recent research progress in measuring human brain activities. Certain measuring methods have been used in this regard, including the implantable microelectrode array (MEA) [1] and other noninvasive measuring methods, such as nearinfrared spectroscopy [2], electroencephalogram [3], functional magnetic resonance imaging (fMRI) [4,5,6,7,8,9,10], and magnetoencephalography (MEG) [11,12]. MEA is an invasive measurement method, and the merit of MEA is its robustness to noise during brain activity measurement. However, it necessitates the implantation of microelectrodes in a subject’s body, which is its demerit, as this imposes a significant physical burden on the subject. Therefore, noninvasive methods, such as fMRI and MEG, which are less likely to directly harm a subject, are more widely used than invasive methods. fMRI in particular is frequently used to measure brain activities and can obtain brain activities with high-spatial resolutions. Compared with MEA, the demerits of fMRI are its sensitivity to noise and the large size of the measurement equipment. MEG is superior to fMRI in terms of temporal resolution and has reasonable spatial resolutions [13,14].

Several positive results have been reported by using machine learning methods to analyze brain activities from these measuring methods. For example, emotion analysis methods have been proposed from brain activities [15,16,17]. Some techniques have been proposed to generate an image caption and reconstruct an image using brain activities while seeing the image [9,18,19,20,21,22]. In addition, image reconstruction is attempted on the basis of brain activities while imagining [20]. Researchers believe that the advancement of machine-learning-based brain decoding will reveal the human brain mechanism. The revelation of the human brain’s mechanism is expected to contribute to a society in which everyone lives comfortably by realizing effective devices that use brain activities as input. For example, a brain–machine interface (BMI) aims for humans to directly operate and communicate with external machines without physical movement, which can assist the daily lives of people with handicaps [23,24,25].

The estimation of visual perception from fMRI data has been actively researched in the field of brain decoding to take advantage of excellent spatial resolutions [6,7,8,26]. fMRI data vary depending on the imaging object [6]. Previous studies [7,26] have attempted to analyze visual perception using classical methods, such as a support vector machine [27] and a Gabor wavelet filter [28]. There is a relationship between visual information extracted from a convolutional neural network (CNN) [29] and fMRI data obtained while seeing an object [8,30]. This relationship suggests that a CNN mimics the visual perception system in the human brain, and visual features extracted by a CNN are essential in estimating visual perception. Previous studies have attempted to estimate CNN-based visual features of images using fMRI data collected while subjects see the images [8,31,32,33,34]. In a previous study [8], the authors constructed a decoder that learns the relationship between each subject’s fMRI data and the visual features of a seen image, and the decoder can estimate the visual features of the seen image from the fMRI data. Their method is based on linear regression. Although this decoder can estimate visual features, the estimation accuracy strongly depends on the size of the training set that consists of fMRI data. However, it is essential to lie in a closed and narrow space for a long period to measure fMRI data. Preparing a large amount of fMRI data also places a psychological and time burden on a subject.

Meanwhile, it is still challenging to estimate visual perception using a limited amount of data. Some studies [31,32] have used multi-subject fMRI data obtained when multiple subjects see the same image. These methods construct the latent space to extract the common features (shared features) from multi-subject fMRI data. These methods are based on the generative model, and they can stably train models using multiple inputs, even though the size of training data is limited. They emphasized the concept of shared features as the common information between multi-subject fMRI data. However, they have not considered individual features on which the researchers [8,35] focused as the subjectspecific information in single-subject fMRI data. Each feature contains different information, and it is possible that accuracy is improved by combining shared and individual features in terms of differences in expressive ability.

In this study, we propose a novel method for estimating the visual features of a seen image from multi-subject fMRI data. To improve the estimation accuracy, we introduce the idea of focusing on multi-subject and single-subject fMRI data. We calculate the latent space based on fMRI data and extract shared and individual features using a generative model. The generative model assumes the distributions of the extracted features and can extract effective features from a limited amount of data. We can train decoders to estimate visual features using the extracted shared and individual features. In addition, it is possible to use common cognitive information from multi-subject fMRI data and subject-specific cognitive information from single-subject fMRI data.

The remainder of this paper is organized as follows. In Section 2, we explain the proposed estimation method using multi-subject fMRI data. Section 3 presents the experimental results using an fMRI dataset when multiple subjects see an image. Finally, Section 4 presents the conclusion.

## 2. Estimation of Visual Features of Seen Image Using Shared and Individual Features

**Figure 1:**
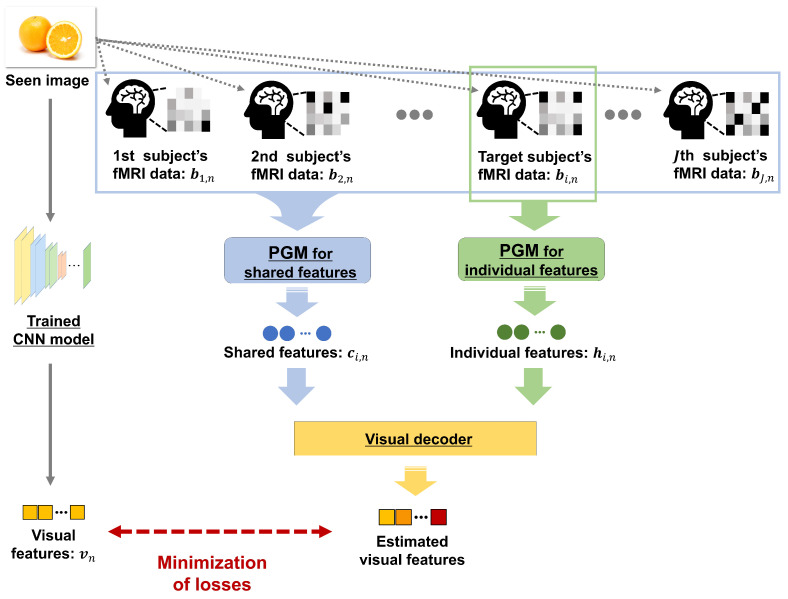
Overview of the training phase in the proposed method. Different PGMs are trained to extract shared and individual features of each subject. We can extract shared features from singlesubject fMRI data. The PGM for individual features uses only single-subject fMRI data as a target for training. The visual decoder was trained using both extracted features from PGMs based on the minimization problem between the estimated and visual features from the trained CNN.

**Figure 2:**
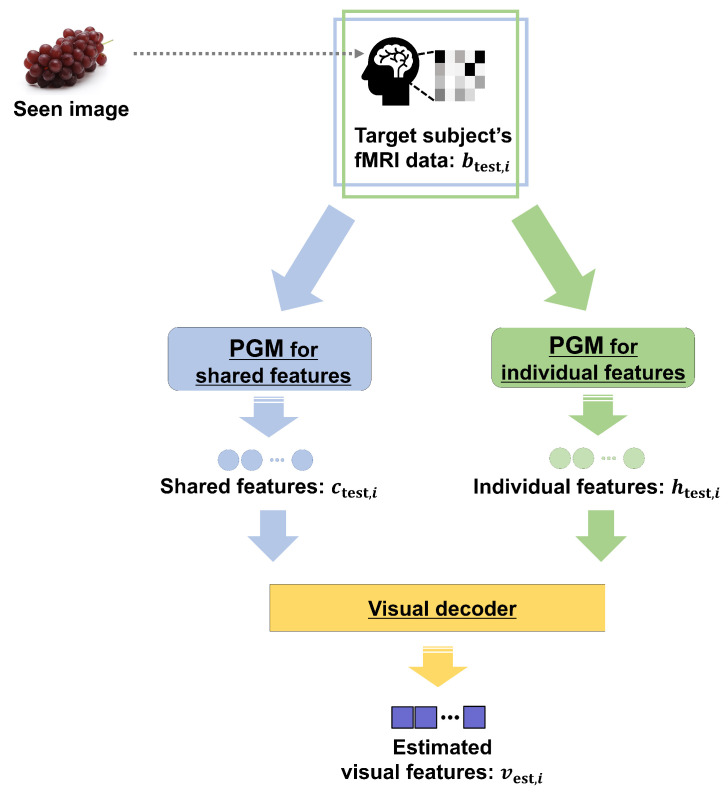
Overview of the test phase in the proposed method. The PGM corresponding to each feature was used to extract features from the target subject’s fMRI data. The trained visual decoder can estimate visual features using shared and individual features, and this scheme realizes our approach.

In this section, we explain the proposed method. The overview of the training phase is illustrated in Figure 1. We constructed a probabilistic generative model (PGM) to extract shared and individual features from multiple and single subjects. In addition, a visual decoder was constructed to estimate visual features from both extracted features. The overview of the test phase is shown in Figure 2. We extracted shared and individual features from single-subject fMRI data using the PGM and estimate the visual features of a seen image using the trained visual decoder. The trained PGM can extract shared features from only single-subject fMRI data. The training and test phases are explained in Section 2.1 and Section 2.2, respectively. A list of the variables used is presented in Appendix A.

### 2.1. Training Phase: Construction of PGM and Visual Decoder

The training phase consisted of two steps. In the first step, we constructed the PGM to extract shared and individual features from fMRI data separately. This model can extract features robust to the noise in fMRI data. In the second step, we trained a visual decoder to estimate the visual features of a seen image. The visual decoder can transform shared and individual features into visual features using a projection matrix.

#### 2.1.1. Step 1: Construction of PGM

In step 1, we constructed the PGM for extracting of shared and individual features from fMRI data $B_{i}=\left[b_{i,1},\cdots ,b_{i,N}\right]\in R^{d_{i}\times N}$ (i=1,…,J; here, *J* represents the number of subjects, $d_{i}$ denotes the dimension of the fMRI data in *i*th subject, and *N* represents the number of fMRI data training corresponding to each seen image). First, we describe the scheme for extracting the shared features $C=\left[c_{1},\cdots ,c_{N}\right]\in R^{d_{\mathrm{com}}\times N}$($d_{\mathrm{com}}$ being the dimensions of the shared features). An algorithm table is shown in Algorithm 1. The Gaussian distribution is introduced as a prior distribution of shared features C into the following minimization problem:

(1) $$\begin{array}{c} \begin{array}{cc} \; & \min_{P_{i},C}\sum_{i=1}^{J}{∥B_{i}-P_{i}C∥}_{F}^{2}\;s.t.\;P_{i}^{⊤}P_{i}=I, \end{array} \end{array}$$

where $P_{i}\in R^{d_{i}\times d_{\mathrm{com}}}$ denotes the projection matrix that transforms the fMRI data $B_{i}$ into the shared features C, and I represents the identity matrix. The prior distribution of the shared features $c_{n}$ (n=1,⋯,N) and the conditional Gaussian distribution $p(b_{i,n}|c_{n})$ are given as follows:

(2) $$\begin{array}{cc} p(c_{n}) & ∼N(0,\Sigma_{c}), \end{array}$$

(3) $$\begin{array}{cc} p(b_{i,n}|c_{n}) & ∼N(P_{i}c_{n}+\mu_{i},\rho_{i}^{2}I), \end{array}$$

where $\Sigma_{c}\in R^{d_{\mathrm{com}}\times d_{\mathrm{com}}}$ denotes the covariance matrix of the shared features $c_{n}$, $\mu_{i}=\frac{1}{N}\sum_{n=1}^{N}b_{i,n}\in R^{d_{i}}$ represents the mean of the fMRI data $B_{i}$, and $\rho_{i}^{2}$ represents the variance of $B_{i}$. The fMRI data of each subject are combined under the assumption that multiple subjects see the same image, and multi-subject fMRI data corresponding to an image $b_{n}={\left[b_{1,n}^{⊤},\cdots ,b_{J,n}^{⊤}\right]}^{⊤}$ are defined. Moreover, the marginal probability distribution $p(b_{n})$ and $b_{n}$ are represented as follows:   

(4) $$\begin{array}{cc} p(b_{n}) & ∼N(\mu ,P\Sigma_{c}P^{⊤}+\Psi ), \end{array}$$

(5) $$\begin{array}{cc} b_{n} & =Pc_{n}+\mu +\epsilon , \end{array}$$

where $P={\left[P_{1}^{⊤},\cdots ,P_{J}^{⊤}\right]}^{⊤}$, $\mu ={\left[\mu_{1}^{⊤},\cdots ,\mu_{J}^{⊤}\right]}^{⊤}$ and $\Psi =\mathrm{diag}\left(\rho_{1}^{2}I,\cdots ,\rho_{J}^{2}I\right)\in R^{d_{\mathrm{all}}}{}^{\times d_{\mathrm{all}}}$ are combined parameters ($d_{\mathrm{all}}=\sum_{i=1}^{J}d_{i})$, and ϵ∼N(0,Ψ) is an error term. To calculate the marginal probability distribution in Equation (4), we define the joint distribution of the shared features $c_{n}$ and fMRI data $b_{i,n}$ and take the logarithm. The mean and covariance matrix of $p(b_{n})$ can be calculated by computing the exponential part of the joint matrix distribution for the second-order and first-order terms [36].
**Algorithm 1:** PGM for shared features $c_{n}$.1:**Initialize:**2:     $P_{i}$, $\rho_{i}$, $\Sigma_{c}$, $\mu_{i}$, *J*, *N*, MAXLOOP, $A_{i}$, $\rho_{i}^{{\mathrm{new}}^{2}}$, ${\Sigma_{c}}^{\mathrm{new}}$3:**for** i=1…J**do**4:    **for** n=1…N **do**5:        $\mu_{i}\leftarrow \mu_{i}+b_{i,n}$6:    **end for**7:    $\mu_{i}\leftarrow \frac{1}{N}\mu_{i}$8:**end for**9:l←110:**while** MAXLOOP≥l**do**            // EM algorithm for updating parameters11:    ${\mathrm{Var}}_{c|b}\left[c\right]\leftarrow \Sigma_{c}-{\Sigma_{c}}^{⊤}P^{⊤}{\left(P\Sigma_{c}P^{⊤}+\Psi \right)}^{-1}P\Sigma_{c}$       // Start expectation step12:    **for** n=1…N **do**13:        $E_{c|b}\left[c_{n}\right]\leftarrow {\left(P\Sigma_{c}\right)}^{⊤}{\left(P\Sigma_{c}P^{⊤}+\Psi \right)}^{-1}\left(b_{n}-\mu \right)$14:        $E_{c|b}\left[c_{n}c_{n}^{⊤}\right]\leftarrow {\mathrm{Var}}_{c|b}\left[c\right]+E_{c|b}\left[c_{n}\right]E_{c|b}{\left[c_{n}\right]}^{⊤}$15:    **end for**                              // End expectation step16:    **for** i=1…J **do**                      // Start maximization step17:        **for** n=1…N **do**18:           $A_{i}\leftarrow A_{i}+\left(b_{i,n}-\mu_{i}\right)E_{c|b}{\left[c_{n}\right]}^{⊤}$19:        **end for**20:        $A_{i}\leftarrow \frac{1}{2}A_{i}$21:        $P_{i}^{\mathrm{new}}\leftarrow A_{i}{\left(A_{i}^{⊤}A_{i}\right)}^{-1/2}$22:        **for** n=1…N **do**23:           $\rho_{i}^{{\mathrm{new}}^{2}}\leftarrow \rho_{i}^{{\mathrm{new}}^{2}}+\left|\right|b_{i,n}-\mu_{i}{\left|\right|}^{2}-2{\left(b_{i,n}-\mu_{i}\right)}^{⊤}P_{i}^{\mathrm{new}}E_{c|b}\left[c_{n}\right]+tr\left(E_{c|b}\left[c_{n}c_{n}^{⊤}\right]\right)$24:        **end for**25:        $\rho_{i}^{{\mathrm{new}}^{2}}\leftarrow \frac{1}{Nd_{i}}\rho_{i}^{{\mathrm{new}}^{2}}$26:    **end for**27:    **for** n=1…N **do**28:        ${\Sigma_{c}}^{\mathrm{new}}\leftarrow {\Sigma_{c}}^{\mathrm{new}}+E_{c|b}\left[c_{n}c_{n}^{⊤}\right]$29:    **end for**30:    ${\Sigma_{c}}^{\mathrm{new}}\leftarrow \frac{1}{N}{\Sigma_{c}}^{\mathrm{new}}$31:    $P_{i}$, $\rho_{i}^{2}$, $\Sigma_{c}$←$P_{i}^{\mathrm{new}}$, $\rho_{i}^{{\mathrm{new}}^{2}}$, ${\Sigma_{c}}^{\mathrm{new}}$                // End maximization step32:    **Initialize:**33:         $A_{i}$, $\rho_{i}^{{\mathrm{new}}^{2}}$, ${\Sigma_{c}}^{\mathrm{new}}$34:    l←l+135:**end while**

We introduce the expectation maximization (EM) algorithm [37] for updating the model parameters $P_{i}$, $\rho_{i}^{2}$, and $\Sigma_{c}$. The posterior distribution $p(c_{n}|b_{n})$ is calculated in the expectation step of the EM algorithm. The posterior distribution $p(c_{n}|b_{n})$ follows the Gaussian distribution, and we can analytically calculate the expected value $E_{c|b}\left[c_{n}\right]$ and the variance ${\mathrm{Var}}_{c|b}\left[c\right]$ as follows:

(6) $$\begin{array}{cc} E_{c|b}\left[c_{n}\right] & ={\left(P\Sigma_{c}\right)}^{⊤}{\left(P\Sigma_{c}P^{⊤}+\Psi \right)}^{-1}\left(b_{n}-\mu \right), \end{array}$$

(7) $$\begin{array}{cc} {\mathrm{Var}}_{c|b}\left[c\right] & =\Sigma_{c}-{\Sigma_{c}}^{⊤}P^{⊤}{\left(P\Sigma_{c}P^{⊤}+\Psi \right)}^{-1}P\Sigma_{c}. \end{array}$$

The expected value $E_{c|b}\left[c_{n}\right]$ and the variance ${\mathrm{Var}}_{c|b}\left[c\right]$ are calculated using the joint distribution of the shared features $c_{n}$ and fMRI data $b_{n}$, similarly to the calculation in Equation (4). However, the joint distribution is defined on the basis of the marginal probability distribution $p(b_{n})$. The joint distribution is taken by logarithm. We calculate the expected value $E_{c|b}\left[c_{n}\right]$ and the variance ${\mathrm{Var}}_{c|b}\left[c\right]$ using the exponential part of the joint matrix distribution for the second-order and first-order terms [36]. Furthermore, the second-order moment $E_{c|b}\left[c_{n}c_{n}^{⊤}\right]$ is calculated as follows:

(8) $$\begin{array}{c} E_{c|b}\left[c_{n}c_{n}^{⊤}\right]={\mathrm{Var}}_{c|b}\left[c\right]+E_{c|b}\left[c_{n}\right]E_{c|b}{\left[c_{n}\right]}^{⊤}. \end{array}$$

The parameters $P_{i}$, $\rho_{i}^{2}$, and $\Sigma_{c}$ are updated to maximize the expected value $R(\theta ,\theta^{\mathrm{old}})$ by ($\theta ,\theta^{\mathrm{old}}∼\left\{P_{i},\rho_{i}^{2},\Sigma_{c}\right\}$) in the maximization step of the EM algorithm. Note that $\theta^{\mathrm{old}}$ is a fixed parameter in the expectation step. The expected value $R(\theta ,\theta^{\mathrm{old}})$ is expressed as follows:

(9) $$\begin{array}{cc} R(\theta ,\theta^{\mathrm{old}}) & =\frac{1}{N}\sum_{n=1}^{N}\int p(c_{n}|b_{n};\theta^{\mathrm{old}})\log p(b_{n},c_{n};\theta )dc_{n}\\ \; & =\frac{1}{N}\sum_{n=1}^{N}\int p(c_{n}|b_{n};\theta^{\mathrm{old}})(\log p(b_{n}|c_{n};\theta )\\ \; & \;+\log p(c_{n};\theta ))dc_{n}\\ \; & =\sum_{n=1}^{N}E_{c|b}[\log p(b_{n}|c_{n};\theta )+\log p(c_{n};\theta )]. \end{array}$$

In the above equation, the transformation is performed with respect to θ. A term with only $\theta^{\mathrm{old}}$ as parameters can be regarded as a constant and is excluded from the expectation value *R* in the maximization step. The expected value $R(\theta ,\theta^{\mathrm{old}})$ is calculated using partial derivatives with respect to the parameters $\left\{P_{i},\rho_{i}^{2},\Sigma_{c}\right\}$ and maximized. The updated parameters $P_{i}^{\mathrm{new}}$, $\rho_{i}^{{\mathrm{new}}^{2}}$ and ${\Sigma_{c}}^{\mathrm{new}}$ are defined as follows:

(10) $$\begin{array}{cc} P_{i}^{\mathrm{new}} & =A_{i}{\left(A_{i}^{⊤}A_{i}\right)}^{-1/2}, \end{array}$$

(11) $$\begin{array}{cc} A_{i} & =\frac{1}{2}(\sum_{n=1}^{N}\left(b_{i,n}-\mu_{i}\right)E_{c|b}{\left[c_{n}\right]}^{⊤}), \end{array}$$

(12) $$\begin{array}{cc} \rho_{i}^{{\mathrm{new}}^{2}} & =\frac{1}{Nd_{i}}\sum_{n=1}^{N}\left(|\right|b_{i,n}-\mu_{i}{\left|\right|}^{2}-2{\left(b_{i,n}-\mu_{i}\right)}^{⊤}\\ \; & \;P_{i}^{\mathrm{new}}E_{c|b}\left[c_{n}\right]+tr\left(E_{c|b}\left[c_{n}c_{n}^{⊤}\right]\right)), \end{array}$$

(13) $$\begin{array}{cc} {\Sigma_{c}}^{\mathrm{new}} & =\frac{1}{N}\sum_{n=1}^{N}E_{c|b}\left[c_{n}c_{n}^{⊤}\right]. \end{array}$$

Each parameter is updated using the expected value $E_{c|b}\left[c_{n}\right]$ and the variance ${\mathrm{Var}}_{c|b}\left[c\right]$ in the expectation step of the EM algorithm. We repeatedly calculated both steps to update the model parameters $P_{i}^{\mathrm{new}}$, $\rho_{i}^{{\mathrm{new}}^{2}}$ and ${\Sigma_{c}}^{\mathrm{new}}$. Finally, the shared features $c_{n}$ can be calculated using the following equation:

(14) $$\begin{array}{c} c_{n}=P^{-1}\left(b_{n}-\mu \right). \end{array}$$

In addition, shared features of each subject can be extracted as follows:

(15) $$\begin{array}{c} c_{i,n}=P_{i}^{-1}\left(b_{i,n}-\mu_{i}\right), \end{array}$$

where $c_{i,n}$ denotes the shared features in the *i*th subject. We can extract shared features following the Gaussian distribution, and the extracted features are effective for estimating visual features due to the robustness of noise.

Similarly, we used the PGM to extract individual features $H_{i}=\left[h_{i,1},\cdots ,h_{i,N}\right]\in R^{d_{\mathrm{ind}}\times N}$ from the fMRI data $B_{i}$ ($d_{\mathrm{ind}}$ being the dimension of the individual features). We also introduce the Gaussian distribution as a prior distribution of the individual features $H_{i}$ into the following minimization problem, and the prior distribution of the individual features $h_{i,n}$ is given as follows:

(16) $$\begin{array}{cc} \; & \min_{P_{i}^{\prime },H_{i}}{∥B_{i}-P_{i}^{\prime }H_{i}∥}_{F}^{2}\;s.t.\;P_{i}^{\prime }{}^{⊤}P_{i}^{\prime }=I, \end{array}$$

where $P_{i}^{\prime }\in R^{d_{i}\times d_{\mathrm{ind}}}$ denotes the projection matrix that transforms the fMRI data $B_{i}$ to the individual features $H_{i}$. The prior distribution of the shared features $h_{i,n}$ and the conditional Gaussian distribution $p(b_{i,n}|h_{i,n})$ are given as follows:

(17) $$\begin{array}{cc} p(h_{i,n}) & ∼N(0,\Sigma_{h_{i}}^{\prime }), \end{array}$$

(18) $$\begin{array}{cc} p(b_{i,n}|h_{i,n}) & ∼N(P_{i}^{\prime }h_{i,n}+\mu_{i},\rho_{i}^{\prime }{}^{2}I), \end{array}$$

where $\Sigma_{h_{i}}^{\prime }\in R^{d_{\mathrm{ind}}\times d_{\mathrm{ind}}}$ denotes the covariance matrix of $h_{i,n}$, and $\rho_{i}^{\prime }{}^{2}$ denotes the variance of $B_{i}$. The marginal probability distributions $p(b_{i,n})$ and $b_{i,n}$ are represented as follows:

(19) $$\begin{array}{cc} p(b_{i,n}) & ∼N(\mu_{i},P^{\prime }\Sigma_{h_{i}}^{\prime }P^{\prime ⊤}+\Psi_{i}^{\prime }), \end{array}$$

(20) $$\begin{array}{cc} b_{i,n} & =P^{\prime }h_{i,n}+\mu_{i}+\epsilon_{i}^{\prime }, \end{array}$$

where $\epsilon_{i}^{\prime }∼N\left(0,\Psi_{i}^{\prime }\right)$ represents an error term. Note that $\Psi^{\prime }=\mathrm{diag}\left(\rho_{i}^{\prime }{}^{2}I\right)\in R^{d_{i}\times d_{i}}$, and $\rho_{i}^{\prime }{}^{2}$ denotes the variance of $B_{i}$. We can extract the individual features $h_{i,n}$ following the calculation steps of the shared features $c_{n}$ using the EM algorithm. The obvious difference between individual features and the shared features $c_{n}$ is that it is not necessary to combine these parameters in multiple subjects. Finally, the individual features $h_{i,n}$ can be calculated using the following equation:

(21) $$\begin{array}{c} h_{i,n}=P^{\prime }{}^{-1}\left(b_{i,n}-\mu_{i}\right). \end{array}$$

#### 2.1.2. Step 2: Construction of Visual Decoder

In step 2, we trained the visual decoder that convert shared and individual features into visual features $V=\left[v_{1},\cdots ,v_{N}\right]\in R^{d_{v}\times N}$ ($d_{v}$ being the dimensions of the visual features). We calculated projection matrices $P_{\mathrm{com},i}\in R^{d_{v}\times d_{\mathrm{com}}}$ and $P_{\mathrm{ind},i}\in R^{d_{v}\times d_{\mathrm{ind}}}$. The following minimization problem was computed with respect to the projection matrices $P_{\mathrm{com},i}$ and $P_{\mathrm{ind},i}$:

(22) $$\begin{array}{cc} \min_{P_{\mathrm{com},i},P_{\mathrm{ind},i}} & ∥V-\left(P_{\mathrm{com},i}C_{i}+P_{\mathrm{ind},i}H_{i}\right){∥}_{F}^{2}\\ \; & +\lambda_{\mathrm{com},i}∥P_{\mathrm{com},i}{∥}_{F}^{2}+\lambda_{\mathrm{ind},i}{∥P_{\mathrm{ind},i}∥}_{F}^{2}, \end{array}$$

where $\lambda_{\mathrm{com},i}$ and $\lambda_{\mathrm{ind},i}$ represent regularization parameters. By repeating the partial derivatives with respect to $P_{\mathrm{com},i}$ and $P_{\mathrm{ind},i}$, we can simply obtain the following optimal projections:

(23) $$\begin{array}{cc} P_{\mathrm{com},i} & =\left(V-P_{\mathrm{ind},i}H_{i}\right){C_{i}}^{⊤}{\left(C_{i}{C_{i}}^{⊤}+\lambda_{\mathrm{com},i}I\right)}^{-1}, \end{array}$$

(24) $$\begin{array}{cc} P_{\mathrm{ind},i} & =\left(V-P_{\mathrm{com},i}C_{i}\right)H_{i}^{⊤}{\left(H_{i}H_{i}^{⊤}+\lambda_{\mathrm{ind},i}I\right)}^{-1}. \end{array}$$

### 2.2. Test Phase: Estimation of Visual Features of Seen Image

The test phase consisted of two steps. In the first step, we extracted shared and individual features using the developed PGM. In the second step, we estimated visual features using shared and individual features using the constructed visual decoder.

#### 2.2.1. Step 1: Extraction of Shared and Individual Features

We extracted the shared features $c_{\mathrm{test},i}$ using the parameters of the PGM as follows:

(25) $$\begin{array}{c} c_{\mathrm{test},i}=P_{i}^{-1}\left(b_{\mathrm{test},i}-\mu_{i}\right), \end{array}$$

where $b_{\mathrm{test},i}\in R^{d_{i}}$ denotes fMRI data for the *i*th subject in the test phase. The shared features could be extracted as each subject’s feature. Similarly, individual features $h_{\mathrm{test},i}$ were extracted as follows:

(26) $$\begin{array}{c} h_{\mathrm{test},i}=P_{i}^{\prime }{}^{-1}\left(b_{\mathrm{test},i}-\mu_{i}\right). \end{array}$$

Both features followed a Gaussian distribution, and we can extract effective features from fMRI data.

#### 2.2.2. Step 2: Estimation of Visual Features

Visual features were estimated using shared and individual features using the trained visual decoder as follows:

(27) $$\begin{array}{cc} v_{\mathrm{est},i} & =P_{\mathrm{com},i}c_{\mathrm{test},i}+P_{\mathrm{ind},i}h_{\mathrm{test},i}, \end{array}$$

where $v_{\mathrm{est},i}$ denotes the visual features estimated using visual decoders in the *i*th subject. In Equation (27), visual features are estimated from shared and individual features using each projection matrix corresponding to the features.

## 3. Experimental Results

This section presents the experimental results of the image category estimation. In Section 3.1, the datasets used in constructing the proposed method are explained. In Section 3.2, the experimental conditions are described. In Section 3.3, the comparison methods are explained. In Section 3.4, the experimental results are presented.

### 3.1. Dataset

In this experiment, we used the fMRI data (approximately 4500-dimensional vectors) published in a previous study [8]. fMRI data comprise data on visual cortex activities of five subjects while observing images with measuring equipment (Siemens MAGNETOM Prisma (https://www.siemens-healthineers.com/jp/magnetic-resonance-imaging/research-systems/magnetom-prisma (accessed on 10 August 2022))). To obtain fMRI data in [8], four males and one female between the ages of 23 and 38 were chosen as the subjects, and the functional localizer [38,39,40] and the standard retinotopy [4,41] experiments were conducted to identify the visual cortex of each subject. There were 1200 seen images of 150 categories collected in ImageNet [42] (eight images per category). We used these images as pairs of fMRI data.

**Figure 3:**
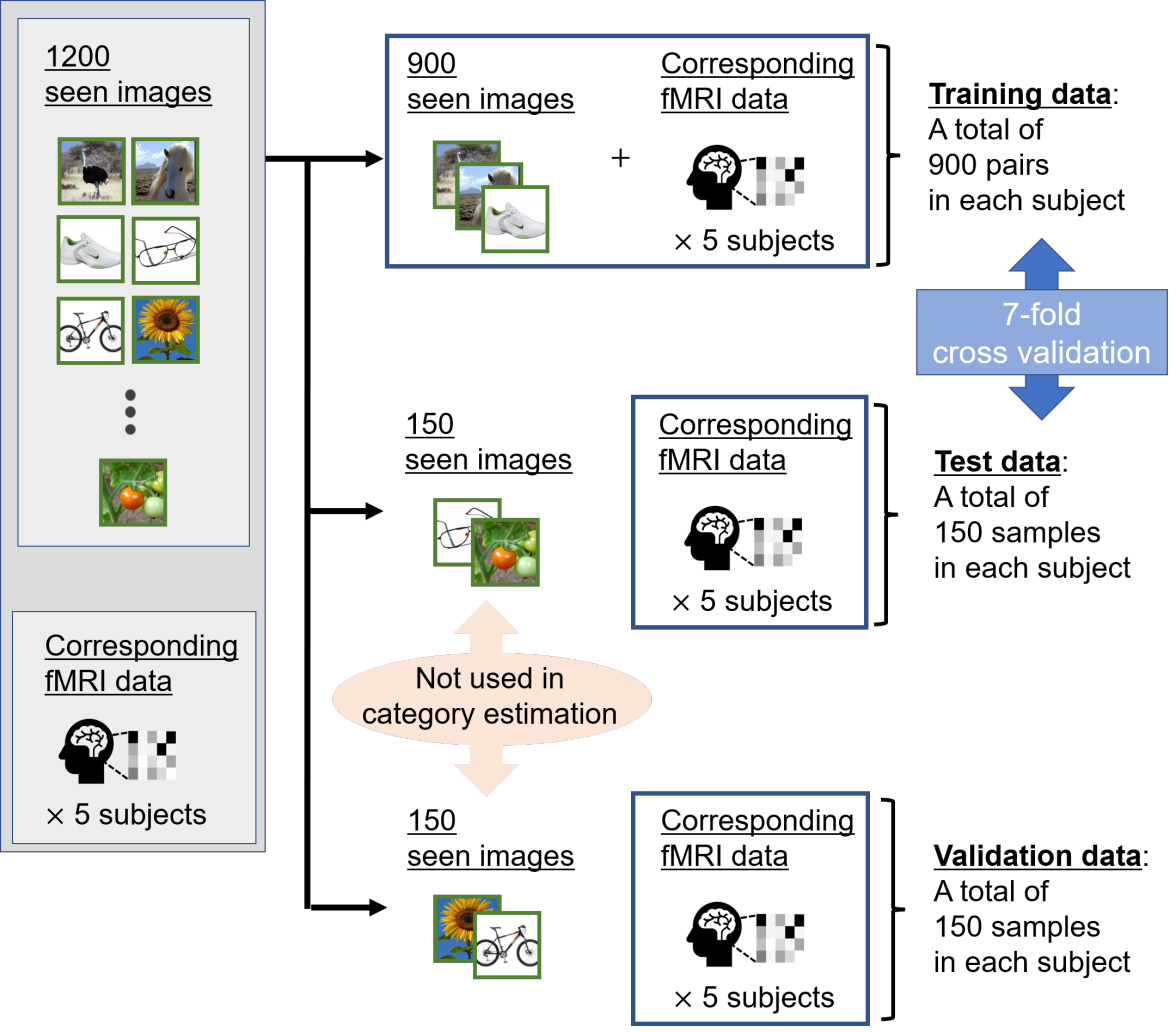
Overview of fMRI datasets of five subjects. We divided a total of 1200 seen images corresponding to measured fMRI data into 900, 150, and 150 images as training, test, and validation data, respectively. The validation data were fixed, and 7-fold cross-validation was applied to 1050 pairs of the training and test data. For category estimation, we used the candidate visual features averaged from other images belonging to the same seen category. Thus, the seen images were not included in the test and validation data.

We performed cross-validation to examine the effectiveness of the proposed method through unbiased experiments. Due to the significant burden on the subject during brain activity acquisition, preparing several samples for the fMRI dataset is difficult. Therefore, as shown in Figure 3, we divided these 1200 pairs into 900, 150, and 150 pairs as training, validation, and test data, respectively. All categories were equally divided into the training, validation, and test data. In addition, we applied 7-fold cross-validation to 1050 pairs consisting of training and test data, respectively. If the training and validation data are interchanged, as is widely done in the machine learning field, the validity of the proposed method on small amounts of test data would be verified.

First, 4096-dimensional visual features were extracted from VGG19 [43]. VGG19 was generally pre-trained for the 1000 categories in ImageNet, and the fully connected layers that extracted visual features were selected farther from the output. Furthermore, the principal component analysis (PCA) [44] was applied to the visual features. The visual features have high dimensions, and we used PCA to prevent overfitting. We selected the cumulative contribution ratio of PCA as 0.8 (the dimensions of visual features applied PCA $d_{v}$, being approximately 70).

### 3.2. Experimental Conditions

**Figure 4:**
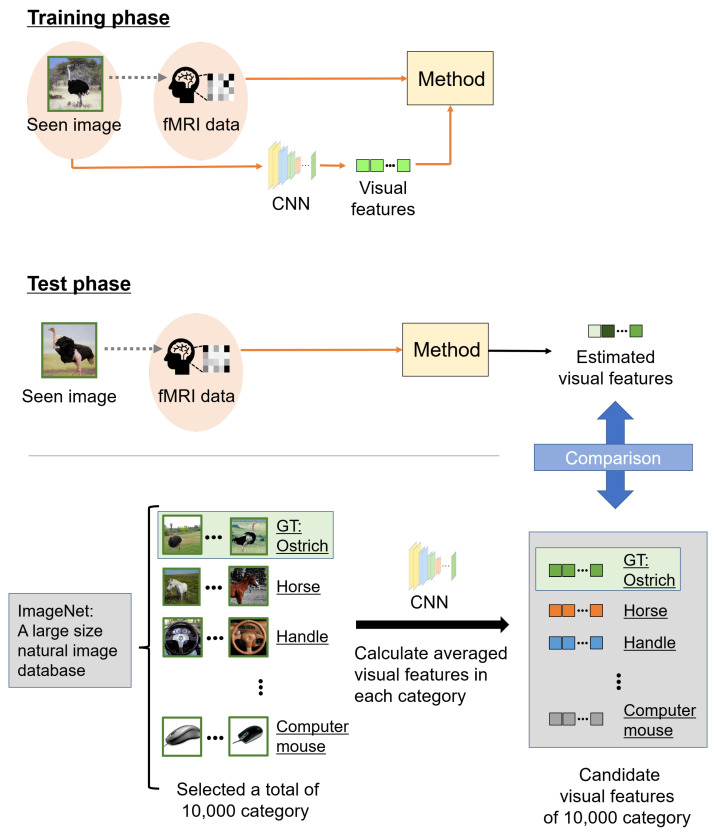
Overview of the scheme of category estimation. In the training phase, the relationship between a seen image and the corresponding fMRI data in each subject was learned. In the test phase, we estimated visual features using fMRI data based on the learned relationship. However, the visual features to be compared were computed from images chosen at random from ImageNet. These other images were 5–10 samples in each category, and we selected 10,000 categories from ImageNet. The 10,000 categories included 150 image categories belonging to the fMRI data in the test phase. We defined candidate visual features, averaged visual features, extracted from these other images in each category, and compared them with estimated visual features from fMRI data. Finally, we calculated the correlations between the estimated and candidate visual features, and the accuracy of the estimations was evaluated with the seen image category as the ground truth (GT).

The estimated accuracy was evaluated by image category estimation. CNNs are mostly trained for image categorization, and category estimation is an appropriate evaluation metric for the representation ability of visual features extracted from a pre-trained CNN. Among the categories of seen images of the fMRI data used in this experiment, some were not used for the pre-trained CNN classifiers. Therefore, we evaluated the estimated accuracy using visual features. Figure 4 shows an overview of category estimation. The image category ranks of the estimated and candidate visual features indicate categories via VGG19 based on the correlations. We selected from 10,000 categories in the fMRI dataset and calculated averaged visual features in each category as candidate visual features. Note that of the 10,000 categories, 150 image categories were used in the fMRI dataset in the test phase. Candidate visual features were 5–10 images chosen at random from each category. The ranks of the estimated visual features and 10,000 candidate visual features were calculated and rearranged in descending order; the ground truth (GT) rank was defined as the image category rank. Finally, the confidence category score *S* was calculated from the image category rank *G* as follows:

(28) $$\begin{array}{c} S=\frac{M-G}{M-1}, \end{array}$$

where *M* represents the total number of image categories, and we set *M* to 10,000 in this experiment. The confidence category score *S* approaches 1 for better image category ranks *G* and 0 for worse. The confidence category scores were averaged in 150 test data points, 7-fold cross-validation sets, and five subjects. We used this metric for the experiment evaluation.

### 3.3. Comparison Methods

We compared the proposed method (hereafter denoted as PM) with several comparison methods (hereafter denoted as CMs) based on the evaluation metric to validate the effectiveness of the PM. The CMs have seven patterns. Two CMs use multi-subject fMRI data and five CMs use single-subject fMRI data.

**Multi-subject** **probabilistic** **generative** **model** **(MSPGM):**

MSPGM is a method based on the PGM, and PM uses multi-subject fMRI data. Visual features are estimated from shared features using ridge regression [45]. We set the number of dimensions in the latent space to the same number of dimensions of the PM and searched for {0.1,1,10} in the regularization parameter of ridge regression.

**Multi-view Bayesian generative model for multi-subject fMRI Data (MVBGM-MS)**:

MVBGM-MS [46] exhibited state-of-the-art performance in the field of brain decoding for visual cognitive contents. MVBGM-MS uses multi-subject fMRI data, and the generative model estimates visual features via the latent space. MVBGM-MS uses visual features, multi-subject fMRI data, and semantic features extracted by inputting image category names into Word2vec [47] to improve accuracy. Therefore, for a fair evaluation of the PM, we used MVBGM-MS without semantic features. We set the number of dimensions in the latent space in the same manner as in the previous study [46].

**Single-subject** **probabilistic** **generative** **model** **(SSPGM)**:

The SSPGM method is based on fMRI data and uses single-subject fMRI data. Visual features are estimated from individual features using ridge regression. We set the number of dimensions in the latent space to the same number of dimensions of the PM and searched for {0.1,1,10} in the regularization parameter of the ridge regression.

**Sparse** **linear** **regression** **(SLR):**

SLR [8] is a baseline method in the field of brain decoding for visual cognitive contents and directly estimated visual features from fMRI data. We estimated visual features by using voxels consisting of fMRI data with a high correlation to the features. Voxels were selected in the order of increasing correlation, and the total number of voxels to be selected was set as a hyperparameter. We searched the number of voxels for {50,100,200,400,500,1000}.

**Canonical correlation analysis (CCA)**:

CCA [48] is a baseline method for calculating the latent space from multi-modal features. Visual features and fMRI data are converted into features belonging to the latent space, and accuracy is evaluated in the space. We searched for $\left\{10,20,30,40,50,d_{v}\right\}$ in the number of dimensions in the latent space.

**Bayesian CCA (BCCA)**:

The BCCA [49] method is an extension of CCA that adopts Bayesian learning. BCCA is a generative model. The latent space consists of visual features and fMRI data, and visual features can be estimated from fMRI data via the space. We searched for $\left\{10,20,30,40,50,d_{v}\right\}$ in the number of dimensions in the latent space.

**Deep CCA (Deep CCA)**:
  The Deep CCA [50] method is also an extension of CCA that adopts deep learning. Similarly to CCA, visual features and fMRI data are converted into features belonging to the latent space, and accuracy is evaluated in the space. We searched for $\left\{10,20,30,40,50,d_{v}\right\}$ in the number of dimensions in the latent space.

### 3.4. Results and Discussion

#### 3.4.1. Estimation Performance Evaluation

Table 1 shows the accuracy of the category estimation in the PM and CMs. Note that the average scores of 150 test images were calculated according to each subject as the evaluation metric. These scores range from 0 to 1, and large values indicate superiority. In the PM, MSPGM, and SSPGM, we set $d_{\mathrm{com}}$, $d_{\mathrm{ind}}$, and the number of iterations in the EM algorithm to 100,100, and 10. In addition, in the PM’s visual decoder, we searched each regularization parameter $\lambda_{\mathrm{com},i}$ and $\lambda_{\mathrm{ind},i}$ for {0.1,1,10}.

In Table 1, the scores, of most subjects and the averages of five subjects in the PM are superior to those in MVBGM-MS and MSPGM based on multi-subject fMRI data. The PM’s superior scores indicate its effectiveness in distinguishing between shared and individual features in multi-subject fMRI data. Furthermore, MVBGM-MS is state-of-the-art, but the PM outperformed it. The scores of all subjects and the averages of the PM are superior to those of SSPGM, SLR, CCA, Deep CCA, and BCCA based on single-subject fMRI data. The SSPGM method is based on the PGM and PM, and its effectiveness in combining shared and individual features is exhibited. The effectiveness of the PM in estimating the visual features of seen images from fMRI data was confirmed compared with SLR, and based on the quantitative accuracy of the PM, it is reliable. Moreover, compared with CCA and Deep CCA, our method can derive their latent spaces successfully. In particular, the PM significantly outperformed Deep CCA, which is the only model that incorporates deep learning among the CMs. Deep CCA is also inferior to simple CCA in terms of score. Deep learning may not be compatible with fMRI data, for which only a small sample size is available. Furthermore, compared with BCCA as a generative model, we can confirm the superiority of our generative model for extracting shared and individual features.

#### 3.4.2. Qualitative Evaluation

**Figure 5:**
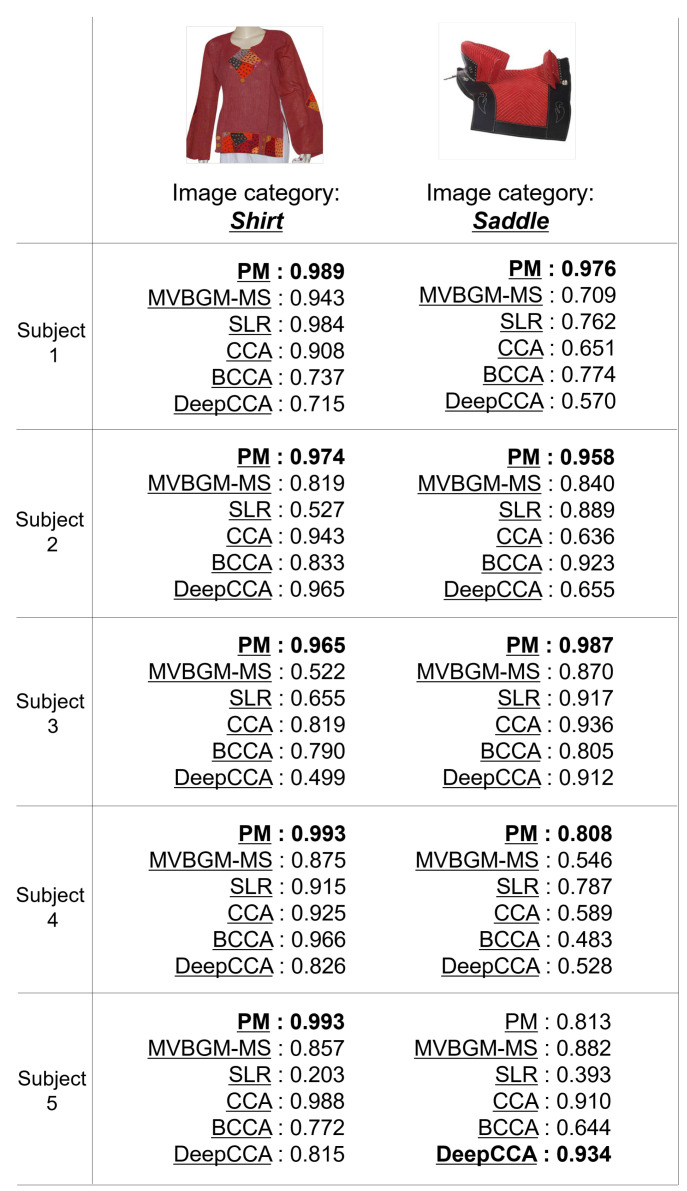
Examples of the category estimation results of PM and five CMs (MVBGM-MS, SLR, CCA, BCCA, and Deep CCA) in the quantitative evaluation. The confidence category scores range from 0 to 1, and the best scores for each subject are shown in bold.

Figure 5 shows the qualitative evaluation of PM and MVBGM-MS, SLR, CCA, BCCA, and Deep CCA, which are methods based on other studies [8,46,48,49,50,51]. For the image categories of “shirt” and “saddle”, the PM has the best confidence category scores for most subjects. These results demonstrate the effectiveness of the PGM as a feature extractor and the idea of using shared and individual features.

**Figure 6:**
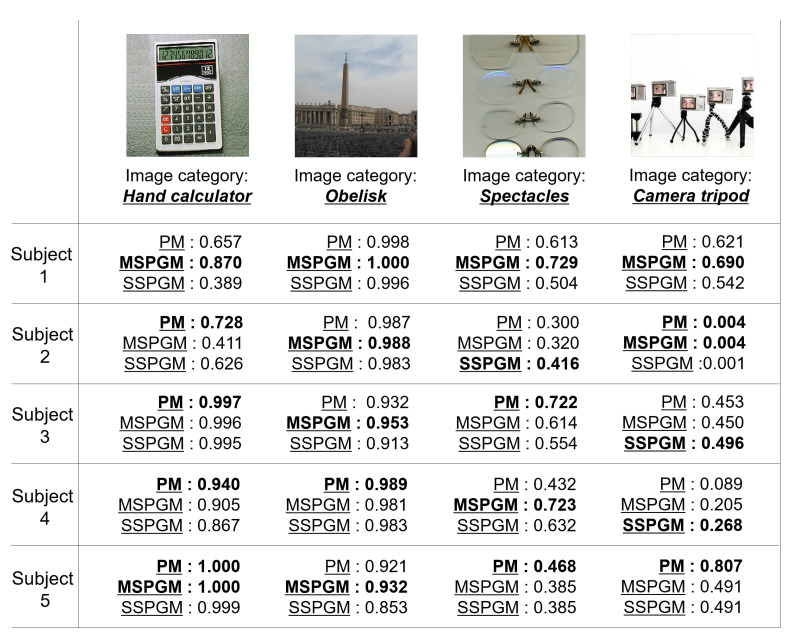
Examples of the category estimation results of PM and two CMs (MSPGM and SSPGM) based on PGM. The confidence category scores range from 0 to 1, and the best scores for each subject are shown in bold.

Figure 6 shows the qualitative evaluation of the PM, MSPGM, and SSPGM based on our PGM. For the image category of “hand calculator”, PM has the best confidence category scores for most subjects. However, for the image category of “obelisk”, MSPGM has the best confidence category scores for most subjects. These results indicate that although the PGM can extract valid shared features, there exists a possibility that it cannot extract individual features. Due to its characteristics, the PGM is superior in extracting shared features from multi-subject fMRI data. For the image categories “spectacles” and “camera tripod”, all methods did not achieve sufficient confidence category scores compared with the quantitative evaluation in most subjects in Table 1. These images contained multiple objects, and a subject’s gaze may not be focused on a single object during fMRI data acquisition. In addition, for the category of “camera tripod”, a part of a human face also appears in the image, which may have affected the subjects’ cognition. Category estimation may still be a difficult task when seeing images containing multiple objects or objects not related to the image categories.

## 4. Conclusions and Future Work

In this article, we proposed a method for estimating visual information from multisubject fMRI data obtained while subjects observed images. The PM estimated visual features using shared features in multi-subject fMRI data and individual features in singlesubject fMRI data. The PGMs were constructed with respect to each feature from fMRI data and used as effective feature extractors. In addition, we constructed the visual decoder using the shared and individual features to estimate visual features. The experimental results verified the effectiveness of the proposed approaches. Although fMRI data tend to contain measured noises and large individual differences compared with other biological activities, such as an eye gaze, this experiment confirmed the effectiveness of combining multi-subject fMRI data. These findings validated the use of machine learning for biological activity analysis with time and physical factor constraints.

Apart from the increase in the sample size due to the expansion of the fMRI dataset, using modalities other than fMRI data may provide a hint as to how to improve the accuracy. In particular, introducing other information that represents an image, such as image captions, is expected to improve the results. For example, some theories suggest estimating visual features directly from fMRI data and caption features using an image captioning model (caption features) or constructing a latent space combining fMRI data and visual and caption features to improve expressive ability. The human brain contains regions specialized for object recognition related to an image category and regions related to lower-order information, such as object color and shape [41]. Although visual features extracted using a CNN contain information specific to image category classification, they may not contain sufficient information, such as an image color and shape. Therefore, the introduction of image captions that can represent the colors and shapes in images is considered an effective method for extracting information related to images in fMRI data obtained while subjects see the images.

## Figures and Tables

**Table 1:** Confidence category scores were averaged for 150 test images and five subjects in the PM and all CMs (The best scores for each subject, and the averages of all subjects are shown in bold).

	Subject1	Subject2	Subject3	Subject4	Subject5	Average
**Proposed Method (PM)**	0.756	0.806	0.867	0.860	0.778	0.813
MSPGM	0.744	0.801	0.857	0.850	0.771	0.805
MVBGM-MS [46]	0.793	0.764	0.832	0.814	0.756	0.792
SSPGM	0.696	0.802	0.859	0.851	0.763	0.794
SLR [8]	0.772	0.734	0.817	0.809	0.711	0.769
CCA [48]	0.706	0.723	0.796	0.782	0.705	0.742
BCCA [49]	0.661	0.762	0.835	0.824	0.740	0.764
Deep CCA [50]	0.622	0.697	0.792	0.755	0.685	0.710

## Data Availability

Publicly available dataset was analyzed in this study. This data can be found here: https://github.com/KamitaniLab/GenericObjectDecoding (accessed on 10 August 2022).

## Appendix A

Table A1 presents a list of the variables used in Section 2.

**Table A1:** List of variables used for the proposed method in the training and test phases corresponds to Section 2.1 and Section 2.2.

Section 2.1.1: Construction of PGM	
$b_{i,n}$	fMRI data corresponding to *n*th image in *i*th subject
$B_{i}$	fMRI data in *i*th subject ($B_{i}=\left[b_{i,1},\cdots ,b_{i,N}\right]$)
$c_{n}$	Shared features corresponding to *n*th image
C	Shared features ($C=[c_{1},\cdots ,c_{N}]$)
$P_{i}$	Projection matrix that transforms fMRI data in *i*th subject into shared features
*I*	Identity matrix
$\Sigma_{c}$	Covariance matrix of shared features
$\mu_{i}$	Mean of fMRI data in *i*th subject
$\rho_{i}^{2}$	Variance of fMRI data in *i*th subject
$b_{n}$	Concatenated fMRI data corresponding to *n*th image for total *J* subjects
μ	Concatenated mean $\mu_{i}$ for total *J* subjects
P	Concatenated projection matrix $P_{i}$ for total *J* subjects
ϵ	Error term of shared features
Ψ	Joint covariance
E	Expected value of expectation maximization (EM) algorithm
Var	Variance of EM algorithm
R	Expected value in maximization step of EM algorithm
${P_{i}}^{\mathrm{new}}$	Updated projection matrix that transforms fMRI data in *i*th subject
$\rho_{i}^{{\mathrm{new}}^{2}}$	Updated variance of fMRI data in *i*th subject
${\Sigma_{c}}^{\mathrm{new}}$	Updated covariance matrix of shared features
$c_{i,n}$	Estimated shared features corresponding to *n*th image in *i*th subject
*J*	Number of subjects
*N*	Number of seen images
*n*	Index of seen images (n=1,…,N)
*i*	Index of subjects (i=1,…,J)
$d_{\mathrm{com}}$	Dimensions of shared features
$d_{i}$	Dimensions of fMRI data in *i*th subject
$d_{\mathrm{all}}$	Sum of dimensions for total *J* subjects
θ	Updated PGM parameters ($\theta ∼\{P_{i}^{\mathrm{new}}$, $\rho_{i}^{{\mathrm{new}}^{2}}$, ${\Sigma_{c}}^{\mathrm{new}}\}$)
$\theta^{\mathrm{old}}$	PGM parameters before update ($\theta^{\mathrm{old}}∼\left\{P_{i},\rho_{i}^{2},\Sigma_{c}\right\}$)
$h_{i,n}$	Individual features corresponding to *n*th image in *i*th subject
$H_{i}$	Individual features in *i*th subject ($H_{i}=\left[h_{i,1},\cdots ,h_{i,N}\right]$)
${P_{i}}^{\prime }$	Projection matrix that transforms fMRI data in *i*th subject into individual features
$\rho_{i^{2}}^{\prime }$	Variance of fMRI data in *i*th subject
${\Sigma_{h_{i}}}^{\prime }$	Covariance matrix of individual features
$\Psi_{i}^{\prime }$	Joint covariance in *i*th subject
$\epsilon_{i}^{\prime }$	Error term of individual features in *i*th subject
$d_{\mathrm{ind}}$	Dimensions of individual features
Section 2.1.2: Construction of visual decoder	
$v_{n}$	Visual features of *n*th image
V	Visual features ($V=[v_{1},\cdots ,v_{N}]$)
$C_{i}$	Estimated shared features in *i*th subject ($C_{i}=\left[c_{i,1},\cdots ,c_{i,N}\right]$)
$P_{\mathrm{com},i}$	Projection matrix that transforms shared features into visual features in *i*th subject
$P_{\mathrm{ind},i}$	Projection matrix that transforms individual features into visual features in *i*th subject
$\lambda_{\mathrm{com},i}$	Regularization parameter corresponding to shared features in *i*th subject
$\lambda_{\mathrm{ind},i}$	Regularization parameter corresponding to individual features in *i*th subject
$d_{v}$	Dimensions of visual features
Section 2.2.1: Extraction of shared and individual features	
$b_{\mathrm{test},i}$	fMRI data in *i*th subject
$c_{\mathrm{test},i}$	Shared features in *i*th subject
$h_{\mathrm{test},i}$	Individual features in *i*th subject
Section 2.2.2: Estimation of visual features	
$v_{\mathrm{est},i}$	Estimated visual features by visual decoder in *i*th subject
